# *Aeromonas hydrophila* RTX adhesin has three ligand-binding domains that give the bacterium the potential to adhere to and aggregate a wide variety of cell types

**DOI:** 10.1128/mbio.03158-24

**Published:** 2025-04-17

**Authors:** Qilu Ye, Robert Eves, Tyler D. R. Vance, Thomas Hansen, Adam P. Sage, Andrea Petkovic, Brianna Bradley, Carlos Escobedo, Laurie A. Graham, John S. Allingham, Peter L. Davies

**Affiliations:** 1 Department of Biomedical and Molecular Sciences, Queen's University 244870, Kingston, Ontario, Canada; 2 Department of Chemical Engineering, Queen's University 562981, Kingston, Ontario, Canada; University of Michigan-Ann Arbor, Ann Arbor, Michigan, USA; VIB/Vrije Universiteit Brussel, Brussels, Belgium

**Keywords:** adhesins, biofilms, protein structure–function, *Aeromonas*, receptor–ligand interaction, binding proteins, antimicrobial agents

## Abstract

Bacteria often make initial contact with their hosts through the ligand-binding domains of large adhesin proteins. Recent analyses of repeats-in-toxin (RTX) adhesins in Gram-negative bacteria suggest that ligand-binding domains can be identified by the way they emerge from “split” domains within the adhesin. Here, using this criterion and an AlphaFold3 model of a 5047-residue RTX adhesin from *Aeromonas hydrophila,* we identified three different ligand-binding domains in this fibrillar protein. The crystal structures of the two novel domains were solved to 1.4 and 1.95 Å resolution, respectively, and demonstrate excellent agreement with their modeled structures. The other domain was recognized as a carbohydrate-binding module based on its beta-strand topology and confirmed by its micromolar affinity for fucosylated glycans, including the Lewis B and Y antigens. This lectin-like module, which was recombinantly produced with its companion split domain and nearby extender domain, bound to a wide variety of cells including yeasts, diatoms, erythrocytes, and human endothelial cells. In each case, 50 mM free fucose prevented this binding and may offer some protection from infection. The carbohydrate-binding module with its neighboring domains also caused aggregation of yeast and erythrocytes, which was again blocked by the addition of free fucose. The second putative ligand-binding domain has a beta-roll structure supported by a parallel alpha-helix, and the third is a homolog of a von Willebrand Factor A domain. These two domains bind to a more limited range of cell types, and their ligands have yet to be identified.

**IMPORTANCE:**

Characterizing the ligand-binding domains of fibrillar adhesins is important for understanding how bacteria can colonize host surfaces and how this colonization might be blocked. Here, we show that the opportunistic pathogen, *Aeromonas hydrophila*, uses a carbohydrate-binding module (CBM) to attach to several different cell types. The CBM is one of three ligand-binding domains at the distal tip of the adhesin. Identifying the glycans bound by the CBM as Lewis B and Y antigens has helped explain the range of cell types that the bacterium will bind and colonize, and it has suggested sugars that might interfere with these processes. Indeed, fucose, which is a constituent of the Lewis B and Y antigens, is effective at 50 mM concentrations in blocking the attachment of the CBM to host cells. This will lead to the design of more effective inhibitors against bacterial infections.

## INTRODUCTION

*Aeromonas hydrophila* is a Gram-negative, rod-shaped bacterium in the family Aeromonadaceae (1, 2). It is widely distributed in aquatic environments, including fresh, brackish, and waste waters. In these ecosystems, the bacterium is typically in sediments and biofilms (3), or associated with other organisms, including plants and animals (4, 5). In humans, diseases caused by *A. hydrophila* include gastroenteritis, wound infections, and septicemia (6, 7). One unusual vector for infections has been the use of leeches in medicine (8). *A. hydrophila* also infects many aquatic animals, causing substantial economic losses in aquaculture (9, 10). Antibiotics and immunization against *A. hydrophila* are two measures used to combat these infections in fish farms (11). However, many strains of *A. hydrophila* are developing resistance against antibiotics (12, 13), posing an emerging threat made more urgent by global warming, which increases the range of this and other water-borne pathogens like *Vibrio cholerae* and *V. vulnificus* (14, 15).

*A. hydrophila* produces a range of diffusible toxins to inhibit competitors and derive nutrients from target organisms (16). Of these, aerolysin, a pore-forming toxin, has been particularly well characterized (17–19). However, little is known about how this bacterium begins colonization at sites of infection. Recent work on the Antarctic marine bacterium *Marinomonas primoryensis*, which binds to sea ice, and on *V. cholerae*, the causative agent for cholera, has implicated long fimbrial adhesins in initiating surface contact with cells through specific ligand-binding domains at the distal end of the adhesins (20, 21). In both of these bacteria, the adhesins are single polypeptide chains of the repeats-in-toxin (RTX) family that are exported through the Type I secretion system but anchored to the bacterial surface by a retention domain that folds internally and is then too large to pass through the channel of the T1SS (22, 23). The RTX long adhesin protein A (LapA) has been well characterized in the beneficial plant bacterium *Pseudomonas fluorescens* (24, 25). Additionally, the characterization of two gene products from the same operon, LapD and LapG, explains how *P. fluorescens* can exit a site of colonization when nutrients like phosphate become growth-limiting (26). LapD is an effector protein that responds to low *c*-di-GMP levels during nutrient starvation by releasing the periplasmic protease LapG to cleave the LapA retention domain, setting the bacterium free to seek a more nutritious environment to colonize.

At the distal, colonizing end of the RTX adhesin, ligand-binding domains determine to what surfaces the bacteria will attach. In *M. primoryensis*, structural analysis of its RTX adhesin, referred to as the *Mp* ice-binding protein (*Mp*IBP), revealed the presence of three ligand-binding domains (27). The largest of the three is the ice-binding domain that can anchor the bacterium to ice (28, 29). Next to it are a PA14-type carbohydrate-binding module (CBM), previously referred to as a sugar-binding domain, with affinity for fucosylated glycans (30), and a peptide-binding domain (PBD) that recognizes and binds a C-terminal tripeptide of optimal sequence -Tyr-Thr-Asp (31). The PA14-type CBM was first characterized in the protective antigen of *Bacillus anthracis* (32) and is widespread in both prokaryotes and eukaryotes (33). The CBM and PBD ligand-binding domains bind to diatoms like *Chaetoceros neogracile* to help form a mutually beneficial mixed-species biofilm under ice where photosynthesis is optimal (27). Similar analyses of the *V. cholerae* flagellar-regulated hemagglutination (FrhA) RTX adhesin responsible for colonizing various organisms and cell types (21) have revealed the presence of two ligand-binding domains. One is a homolog of the PBD from *M. primoryensis* with 65% sequence identity (34). The other is a different type of CBM with less than 20% sequence identity to *Mp*IBPCBM that can bind blood group glycans but is also capable of lysing erythrocytes (35).

Here, we have examined the large RTX adhesin protein of *A. hydrophila* (*Ah*Lap) by AlphaFold3 to predict the structures of all the domains that make up this 0.5-MDa protein. Not only does this modeling correctly predict the start and end of each domain, it can reliably reveal the presence of ligand-binding domains based on their projection out of companion “split domains” (36). By these criteria, there are three C-terminal ligand-binding domains in *Ah*Lap. We expressed each of these as recombinant proteins and solved the crystal structures of two. One is a carbohydrate-binding module with affinity for fucosylated glycans that allows it to bind to a wide range of cells, from yeasts to human endothelial cells and erythrocytes. The other two domains have a much more restricted range of targets. By characterizing these domains and elucidating their binding sites and ligands, antagonists can potentially be developed to block binding. This strategy may aid in inhibiting colonization and infection, particularly with antibiotic-resistant strains.

## RESULTS

### A 0.5-MDa RTX adhesin in *A. hydrophila* with over 40 domains

**Fig 1:**
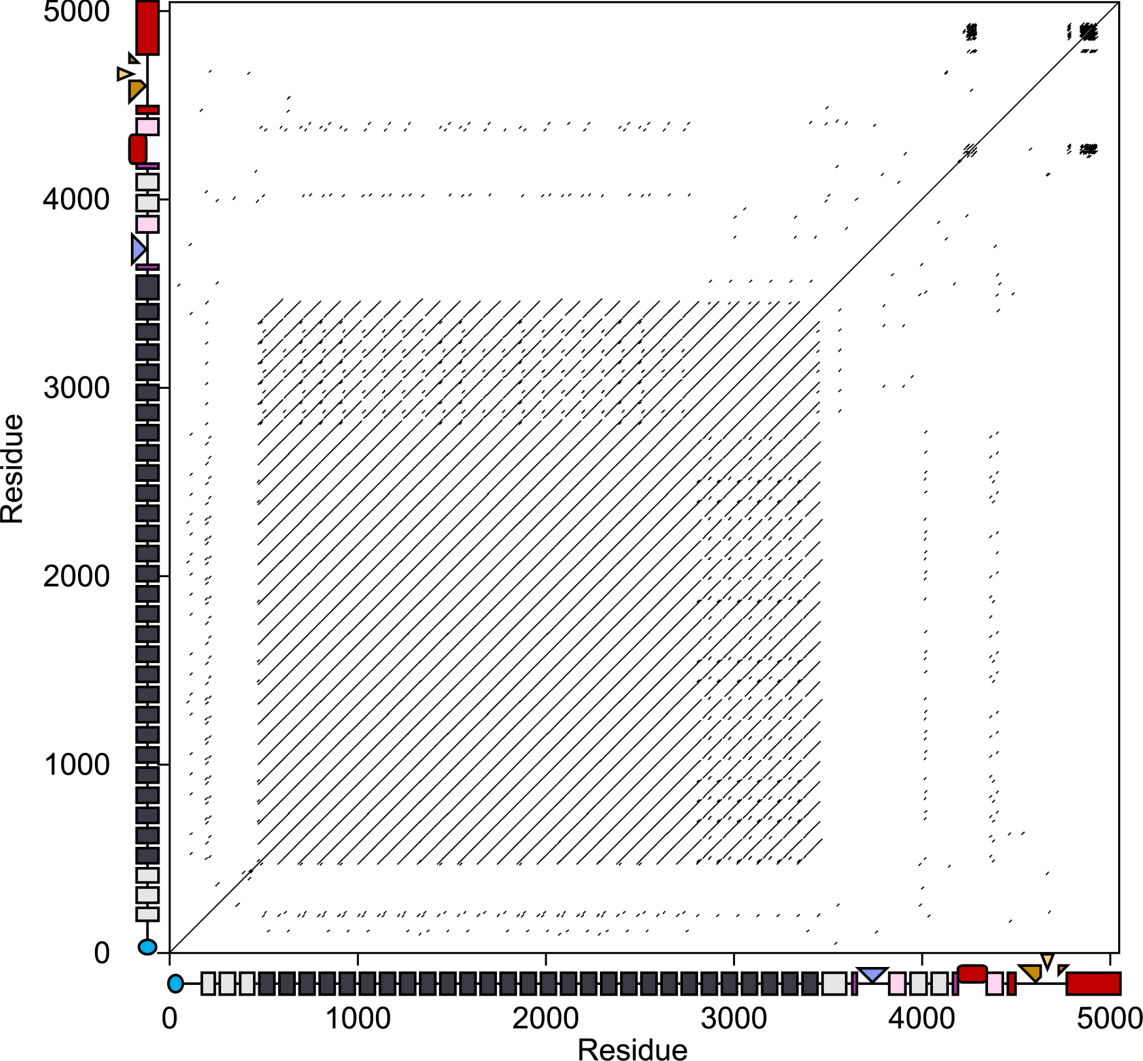
Dot matrix analysis of *Ah*Lap against itself to detect repetitive regions. The *x*- and *y*-axes display the length of *Ah*Lap in amino acid residues alongside domain maps of the adhesin. The N-terminal retention domain is in blue, followed by grey and black rectangles representing the extender domains. In the ligand-binding region, Ig-like split domains are in pink, and the projected CBM, RTX domain, and vWFA domain are colored slate blue, dark red, and gold, respectively. The vWFA domain emerges from the C-terminal RTX domain (red). Dashes in the two-dimensional plot record matches with a threshold score of at least 30 using the BLOSUM62 matrix with a window size of 10 residues (37).

**Fig 2:**
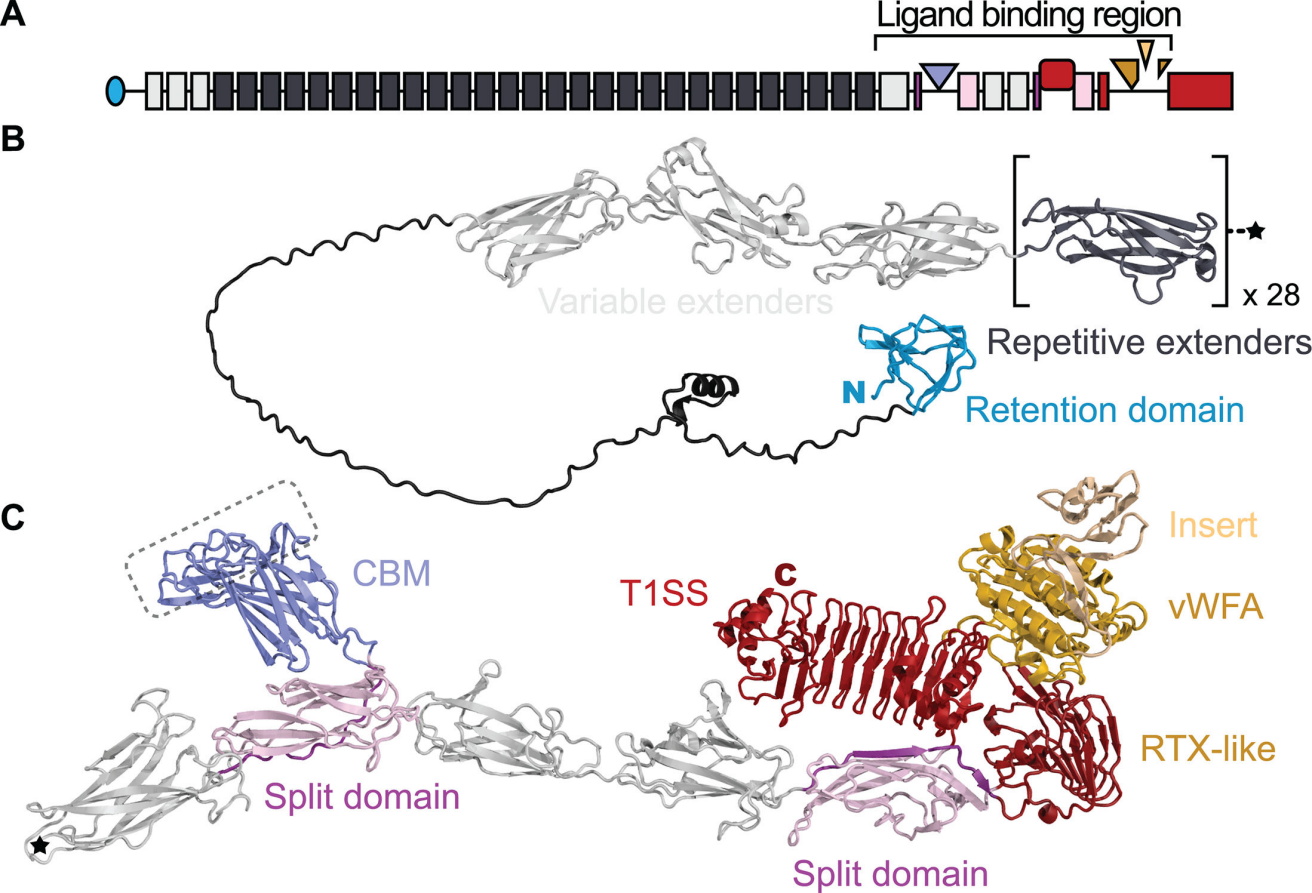
AlphaFold3 structure of *Ah*Lap in ribbon format in relation to the adhesin domain map. (**A**) Domain map of *Ah*Lap as presented in Fig. 1. (**B**) Structure of the retention domain (blue) and extender (light and dark grey) region of the adhesin. The square brackets around the dark grey extender domain indicate that there are 28 highly similar versions of this fold. The star denotes the end of the repetitive extender region. (**C**) Structure of the remainder of the *Ah*Lap adhesin from the star to the C-terminal end. The domains here in the ligand-binding region are colored as described in the Fig. 1 legend. The putative ligand-binding site on the CBM is indicated by the dashed line boundary. Note that the insert in the vWFA domain is colored yellow to distinguish it from the main section (gold). The N and C termini of these two adhesin halves are marked N in blue and C in red, respectively.

RTX adhesins are typically encoded by the largest or second-largest open reading frame within the genomes of many Gram-negative bacteria. A 15-kb open reading frame in the *A. hydrophila* genome encoded a 5047-residue protein identified as an RTX adhesin using dot matrix analysis (Fig. 1). The plot showed the classic signature of an RTX adhesin, where residues in the first two-thirds of the sequence (from 482 to 3455) form a long stretch of tandemly repeated immunoglobulin-like (Ig-like) extender domains that present as an array of equally spaced lines parallel to the central diagonal line. In addition, the three tight clusters of shorter repeats forming black patches on the diagonal in the top right-hand corner come from the Ca^2+^-binding nonapeptide repeats that form the beta-rolls and inspired the name “repeats-in-toxin”. The two clusters near the C terminus correspond to a discontinuous beta-helix that lies immediately before the Type I secretion signal (Fig. 2). The third corresponds to a beta-roll that is one of the three ligand-binding domains.

When this adhesin was modeled by AlphaFold3, a total of 42 discrete domains could be distinguished (Fig. 2). The N-terminal retention domain (blue) had the same Ca^2+^-independent fold seen in the ice-binding adhesin of *M. primoryensis* (27), in LapA of *P. fluorescens* (22), and in the FrhA adhesin of *V. cholerae* (34). The first three extender domains (light grey in Fig. 1, 2A and B) are distinct from the following more highly repetitive extender domains (dark grey in Fig. 1, 2A and B), which is why their repeat signature of lines parallel to the diagonal is muted. A similar observation was made with the ice-binding adhesin of *M. primoryensis*, where the first three domains were varied in their sequences, while subsequent Ig-like extender domains were identical (27).

### The ligand-binding region of *Ah*Lap has three putative ligand-binding domains

**Fig 3:**
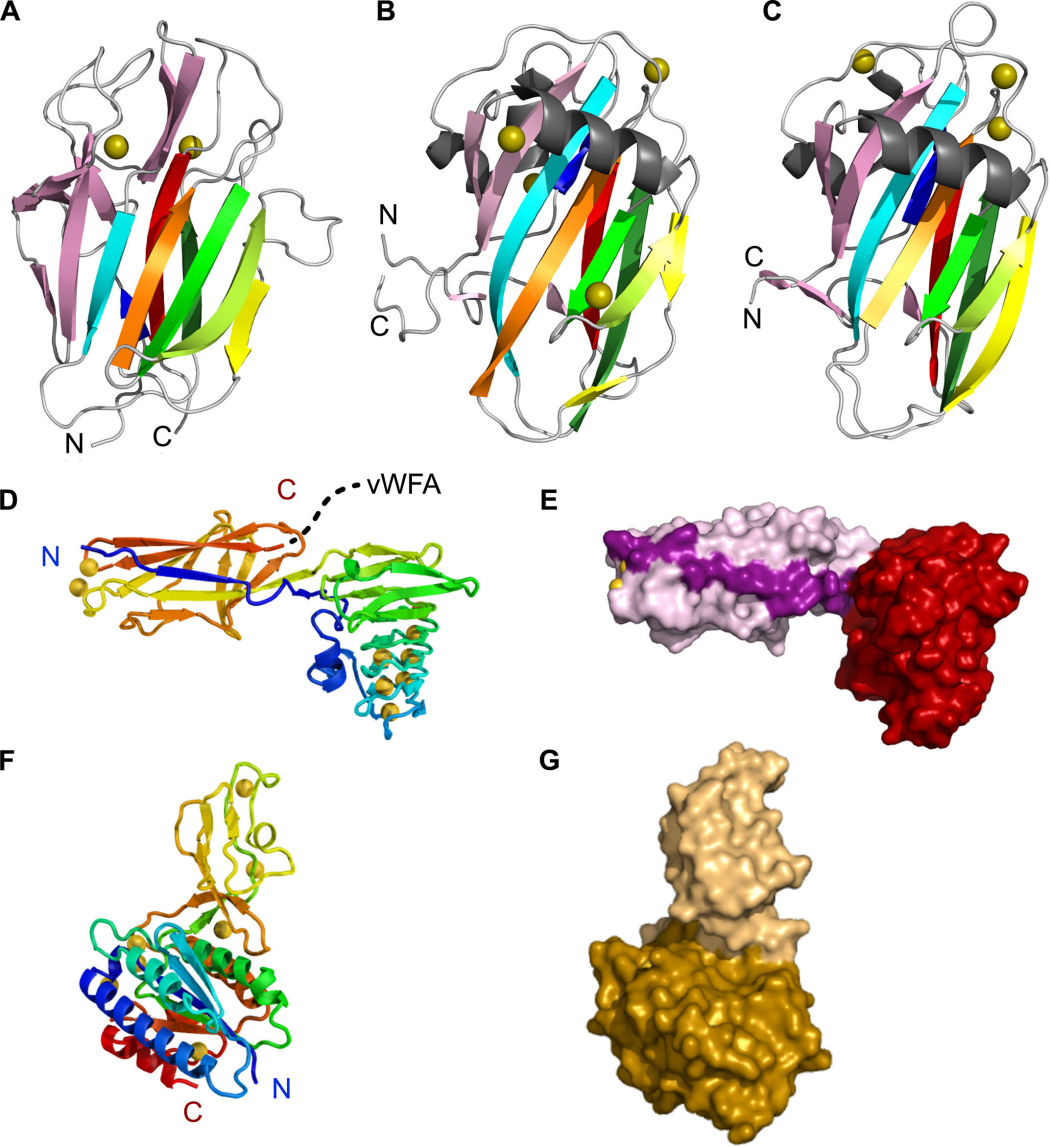
Structures of the three *Ah*Lap ligand-binding domains. (**A**) The AlphaFold3 model of *Ah*LapCBM is shown as a nine-stranded beta-sandwich fold made up of a four-stranded antiparallel beta-sheet on one side and a five-stranded antiparallel beta-sheet on the other side. (**B**) Crystal structure of the CBM from *Mp*IBP (PDB: 5J6Y) showing the similarity of its beta-strand topology (30). (**C**) Crystal structure of the CBM from *Mn*Lap (PDB: 6M8M), again showing the similarity of its beta-strand topology (38). (**D**) Crystal structure of the RTX-like two-domain construct (from residue 4168 to 4498) in rainbow ribbon format that traces the path of the polypeptide chain through the two domains. Numerous Ca^2+^ ions (gold spheres) are present, especially in the RTX solenoid domain. The dotted line from the C terminus leads into the vWFA domain. (**E**) Space-filling presentation of the RTX-like two-domain construct. The split domain strand that leads into the RTX domain is colored a darker pink than the rest of the split domain. (**F**) Crystal structure of one vWFA molecule from the crystal asymmetric unit. The rainbow ribbon tracing shows how the insert sequence buds out of the Rossman fold before the latter is complete. (**G**) Space-filling mode of the vWFA structure, with the main section corresponding to the Rossman fold colored gold and the insert sequence that is missing from some other vWFA domains colored beige.

Immediately after the tandem extender domains is the more varied ligand-binding region (Fig. 2C). Following the last, slightly larger extender domain (leftmost light grey domain in Fig. 2C) is an Ig-like split domain, with the N-terminal portion of the domain in purple, and the portion that is C-terminal to the ligand-binding domain in pink. These split domains serve to project a ligand-binding domain away from the axis of the adhesin, with its ligand-binding site farthest from the junction with the split domain (34). Here, this first ligand-binding domain (colored slate blue in Fig. 2A and C) shares beta-strand topology and low sequence identity with other carbohydrate-binding modules found in bacterial adhesins (Fig. 3A through C) (30, 38). This unit is followed by two Ig-like extender domains (light grey) that lead into a second split domain (purple and pink). Budding out from this second split domain is a 17-kDa putative ligand-binding domain (dark red), which AlphaFold3 suggests is an RTX-type beta-roll containing nonapeptide Ca^2+^-binding sequences. After the fold of the second split domain is completed, the polypeptide chain leads into the C-terminal RTX beta-roll domain (red). This solenoid structure also serves as a split domain from which a von Willebrand Factor A (vWFA) domain emerges (bronze). Finally, the von Willebrand Factor A domain itself serves as a split domain to bud off an insertion sequence (peach) before completing its fold and returning to the beta-roll domain that is followed immediately by the Type I secretion signal, containing short alpha-helices, that marks the C-terminal end of the adhesin.

### Structures of the ligand-binding domains

#### Carbohydrate-binding module

To study the carbohydrate-binding module (CBM) of *Ah*Lap, a 49,630-Da, three-domain construct (CBM_3_) that included this module, along with its split domain and the preceding extender domain (Asn3455 to Gly3940), was produced in *Escherichia coli* from a synthetic gene as a His-tagged recombinant protein (colored light grey, pink, and slate blue in Fig. 2C and corresponding to the three N-terminal domains). CBM_3_ was purified by nickel affinity chromatography, size-exclusion chromatography, followed by another round of nickel affinity chromatography, and stored in aliquots at −80°C at a concentration of 23 mg/mL.

During size exclusion chromatography on a Superdex column, *Ah*LapCBM_3_ eluted as a monomer with an apparent molecular weight of ~50 kDa (Fig. S1A). To check for binding interaction with the dextran-based column matrix, the chromatography was repeated with 10 mM glucose in the column buffer, and the elution profile (red line) gave the same apparent molecular weight from which we concluded that CBM_3_ is a monomer in solution.

When the modeled structure of the putative *Ah*LapCBM (Fig. 3A) is compared to the crystal structures of the PA14 CBMs of *M. primoryensis* IBP (30) and *Marinobacter nauticus* (formerly *Marinobacter hydrocarbonoclasticus*) (38), their beta-strand topologies are identical. This is a key observation given that *Ah*LapCBM and *Mp*CBMs share only 20% sequence identity, and *Ah*CBM and *Mn*CBMs have 23% sequence identity. In contrast, the *Mp*CBM and *Mn*CBMs share 40% sequence identity. At the outer edge of the CBMs is a set of peptide loops that connect the strands of the core beta-sandwich fold. This region in *Ah*LapCBM is the presumed ligand-binding site (circled in Fig. 2C) based on a comparison with the *Mp*CBM and *Mn*CBMs, where co-crystal structures show the presence of a bound Ca^2+^ ion helping coordinate their sugar ligands (30, 38).

#### RTX-like ligand-binding domain

Immediately C-terminal to the split domain from which the CBM buds are two regular extender domains (grey in Fig. 2C). After these, another split domain begins (colored pink) that leads into a second putative ligand-binding domain (colored dark red) before returning to finish the split domain (pink). These two domains were expressed together, purified, and crystallized (Fig. 3D and E). Their combined structure (PDB ID: 9CSE) was solved by collecting data from Ho^3+^-soaked crystals and then using this solution to determine a 1.95 Å-resolution structure from a native data set (Table S1). In the rainbow-colored ribbon presentation (Fig. 3D), the polypeptide chain continues on from the first beta-strand of the split domain (colored blue) to form the ligand-binding domain (colored blue to green). The first section of the beta-roll domain is a short alpha-helix that runs parallel with, and leads into, a short beta-solenoid domain composed of tandem RTX nonapeptide repeats with a consensus sequence X-G-G-X-G-(N/D)-D-X-(L/I/F)-X that together bind nine Ca^2+^ ions on the inside of the solenoid. After four coils of the beta-roll, the domain ends in a short beta-sandwich motif before the polypeptide chain returns to complete the split domain (colored yellow to red). There is no obvious ligand-binding site on this domain, but based on observations of other split + ligand-binding domain combinations (34), its binding site is likely to be the most solvent-exposed surface. In the space-filling presentation (Fig. 3E), the split domain is colored pink with the strand leading into the beta-roll domain (red) shown in a darker pink.

#### vWFA domain with an insertion sequence

Immediately following the second Ig-like split domain lies the main RTX beta-roll that ends in the C-terminal Type I secretion signal. But, after just two turns of this beta-roll domain, the polypeptide chain diverges to fold into a vWFA domain (Fig. 2C). This 28.3-kDa domain was produced as a His-tagged recombinant protein and purified for crystallography. Its crystal structure (PDB ID: 9DAS) was solved for the Ca^2+^-bound protein on our home X-ray source (39). Subsequently, the structure resolution was improved to 1.4 Å with a data set collected at the APS synchrotron (Table S2). The rainbow-colored ribbon presentation (Fig. 3F) shows how the N and C termini (blue and red, respectively) lie adjacent to each other, which is a typical feature of ligand-binding domains because they bud out of their companion split domains. The vWFA domain has a classic Rossman fold of which the first half (in red and orange) is laid down before a 9-kDa insertion sequence (yellow and light green) emerges around the mid-point between residues 4628 and 4704 in the full-length adhesin. This insertion sequence folds into a short alpha-helix and an antiparallel beta-sheet with at least one Ca^2+^ ion present that coordinates OD1 and OD2 of Asp4647 and carbonyl of Arg4641. The polypeptide chain returns to complete the C-terminal portion (residues 4709–4773) of the Rossman fold colored green and blue. It is not known if the insert, colored peach in the space-filling presentation (Fig. 3G), modifies the binding specificity of the vWFA domain (colored gold) or if it is a ligand-binding domain on its own. In the latter case, the vWFA domain would be its split domain. In the crystal structure, there are two molecules of the vWFA domain in the asymmetric unit, with two salt bridges formed between molecule B Arg4641 NE1 and NE2 and molecule A Asp4635 CD1, respectively, as well as seven hydrogen-bond contacts on the surface between the insertion regions of molecules A and B. However, when the PDBePISA program (40) was used to evaluate the macromolecular interface between vWFA domains, the complexation significance score was zero on a scale of 0–1, suggesting that the vWFA domain only makes protein–protein contacts in the crystal. Indeed, the apparent molecular weight of the vWFA domain during size-exclusion chromatography is consistent with it being a monomer in solution (Fig. S1B).

#### Crystal structures of the ligand-binding domains closely match AlphaFold3 predictions

When the crystal structures of the two putative ligand-binding domains (red and gold ribbon) are overlaid on their AlphaFold3 models (green ribbon) to compare their folds (Fig. S2A and B), the agreement between individual domains is extremely close, with RMSD values of below 0.5 Å (Table S4). Deviations are slightly higher in the loops and at the termini.

### *Ah*LapCBM_3_ binds fucosylated glycans

**Fig 4:**
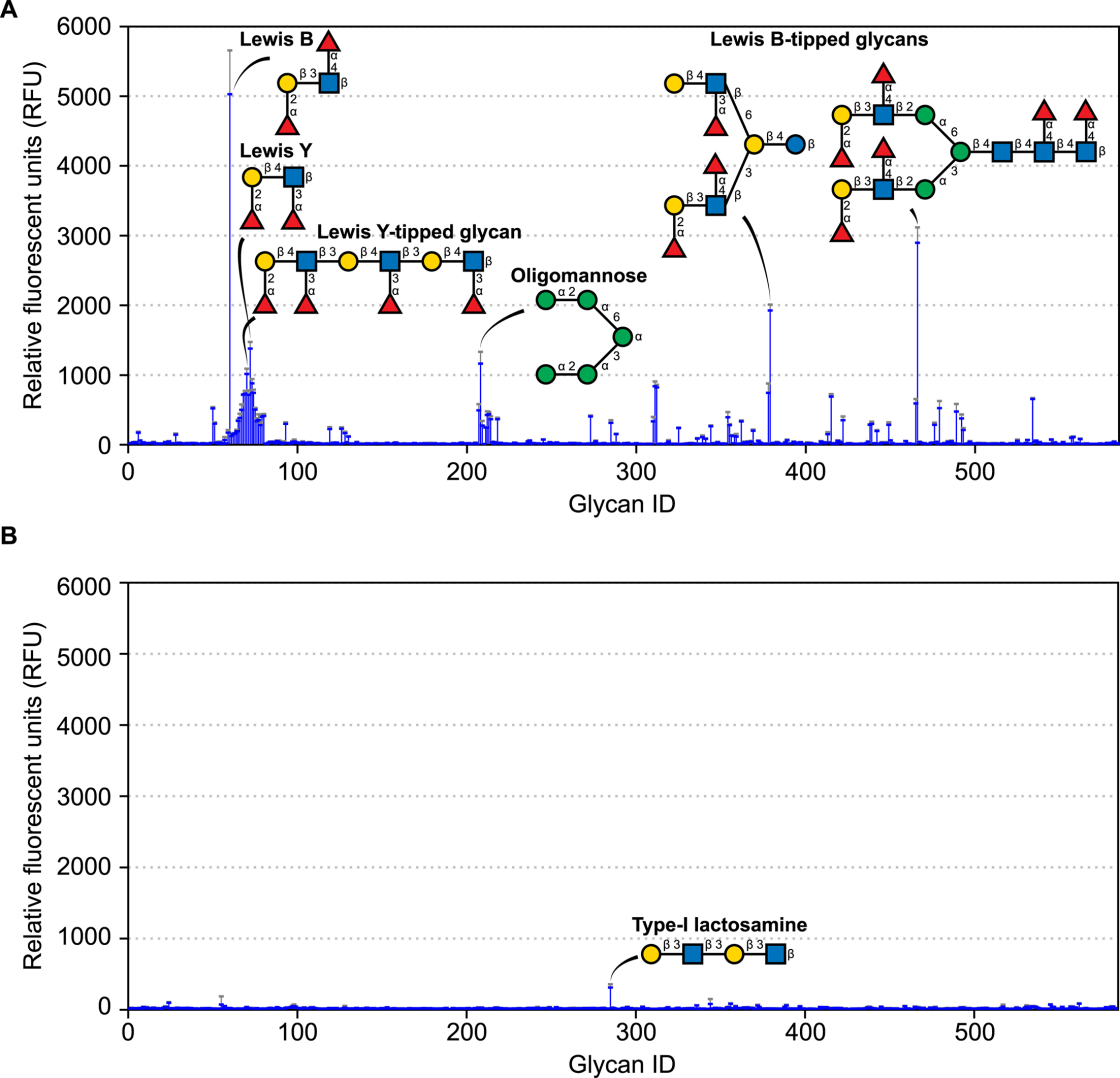
Glycan array analysis. (**A**) Analysis using GFP-tagged *Ah*LapCBM_3_ to probe 580+ mammalian glycans. Binding is shown in blue as relative fluorescence units with the standard deviation from four replicates indicated by error bars above the peaks. Six of the main glycans bound are shown in schematic representation where the sugar moieties are fucose (red triangles), galactose (yellow circles), glucose (blue squares), and mannose (green circles). (**B**) Analysis using GFP-tagged *Ah*LapRTX domain showing the glycan structure of an interactor that is marginally above background levels.

One of the challenges of studying the ligand-binding domains of bacterial adhesins lies in determining what molecules they bind. Putative CBMs can be assayed using glycan arrays. To prepare for this analysis, the *Ah*LapCBM_3_ construct shown in Fig. 3A was labeled at its N-terminal end by fusion to green fluorescent protein (GFP). This CBM_3_ bound avidly to a small set of glycans on the array (Fig. 4A). Strong binding was observed to the Lewis B and Lewis Y antigens as well as to more complex glycans that were tipped with these antigens. These glycans all share terminal fucose residues. Binding was also seen to a branched oligomannose.

**Fig 5:**
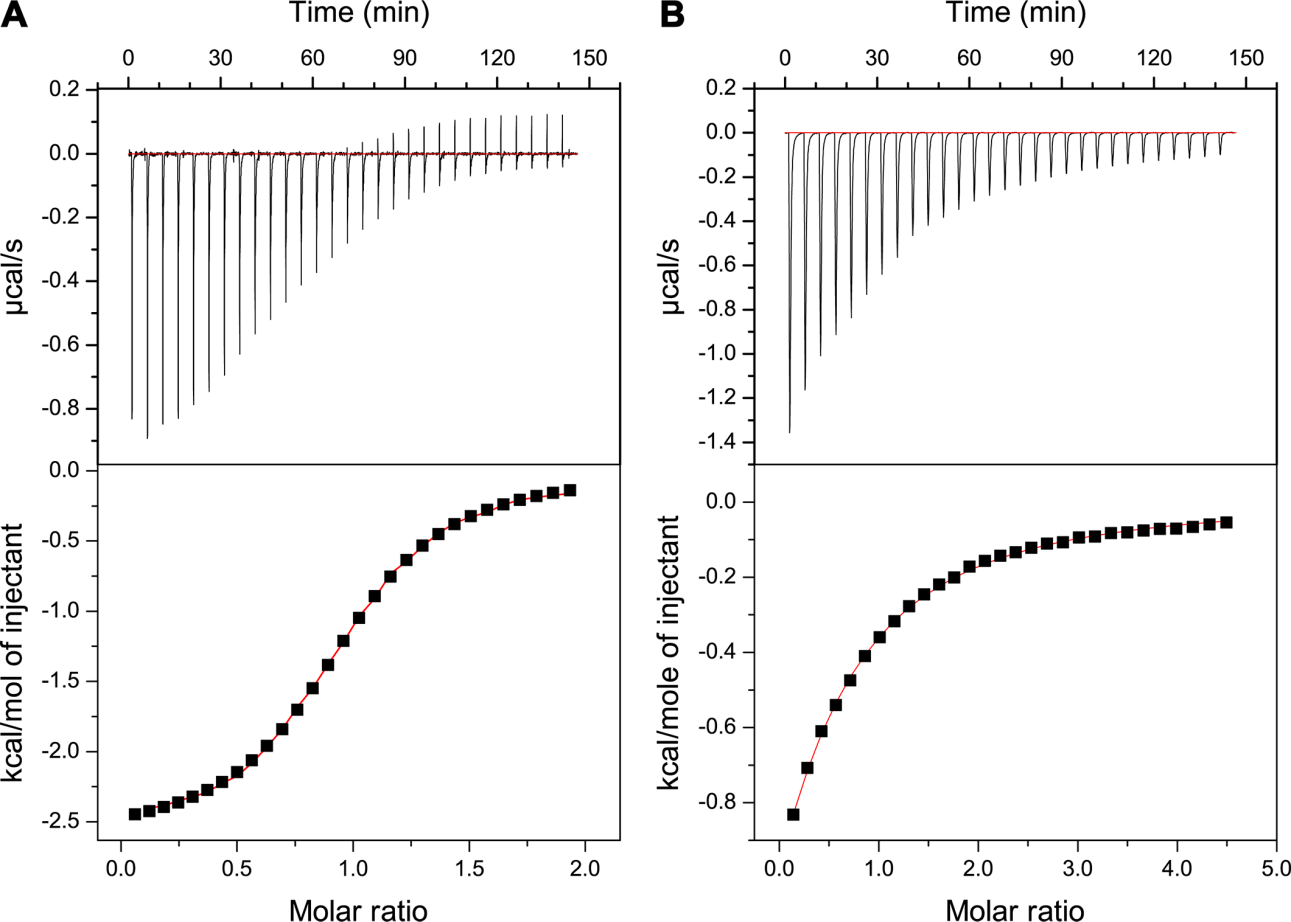
Isothermal titration calorimetry. (**A**) Analysis of *Ah*LapCBM_3_ binding Lewis B antigen. Top panel shows the thermogram for the interaction between Lewis B antigen and *Ah*LapCBM_3_ plotted as µcal/s per event. The lower panel shows the fitted curve of these data from which the stoichiometry and *K*_D_ were calculated. (**B**) Analysis of *Ah*LapCBM_3_ binding free fucose. Top panel shows the thermogram for the interaction between l-fucose and *Ah*LapCBM_3_ plotted as µcal/s per event. The lower panel shows the fitted curve of these data from which the *K*_D_ was calculated.

Isothermal titration calorimetry was used to measure the stoichiometry and strength of binding of the Lewis B antigen to *Ah*LapCBM_3_. When the ligand concentration was fixed at 3.5 mM and the protein concentration was varied between 170 and 467 µM, the stoichiometry of binding was 1:1, and the average *K*_D_ value of five measurements was 20.8 µM (Fig. 5A). The stoichiometry and *K*_D_ value are in line with values obtained for the CBMs of *V. cholerae*, *Aeromonas veronii*, and *M. primoryensis* when tested with fucose as the ligand (30, 35). However, when free fucose was assessed as the ligand for *Ah*LapCBM_3_, its *K*_D_ value of ~386 µM ± 15.2 µM showed much weaker binding for this simple sugar (Fig. 5B). This suggests that other elements of the Lewis B antigen besides the fucosyl moiety contribute to the glycan binding.

### The putative RTX-like ligand-binding domain does not bind mammalian glycans

The binding partner for the RTX-like domain (*Ah*LapRTX) is not known. To determine if this domain is another type of CBM, it was also tagged with GFP at its N terminus and sent for glycan array analysis. It bound to only one glycan slightly above background levels (Fig. 4B). This was galactose-tipped Type-I lactosamine. Therefore, we do not consider it to be a glycan-binding domain. vWFA domains in animals function in binding cells and extracellular matrix proteins like collagen (41, 42). For this reason, the *Ah*LapvWFA domain linked to GFP was not sent for glycan binding analysis.

### *Ah*LapCBM_3_ has affinity for a variety of human cells

**Fig 6:**
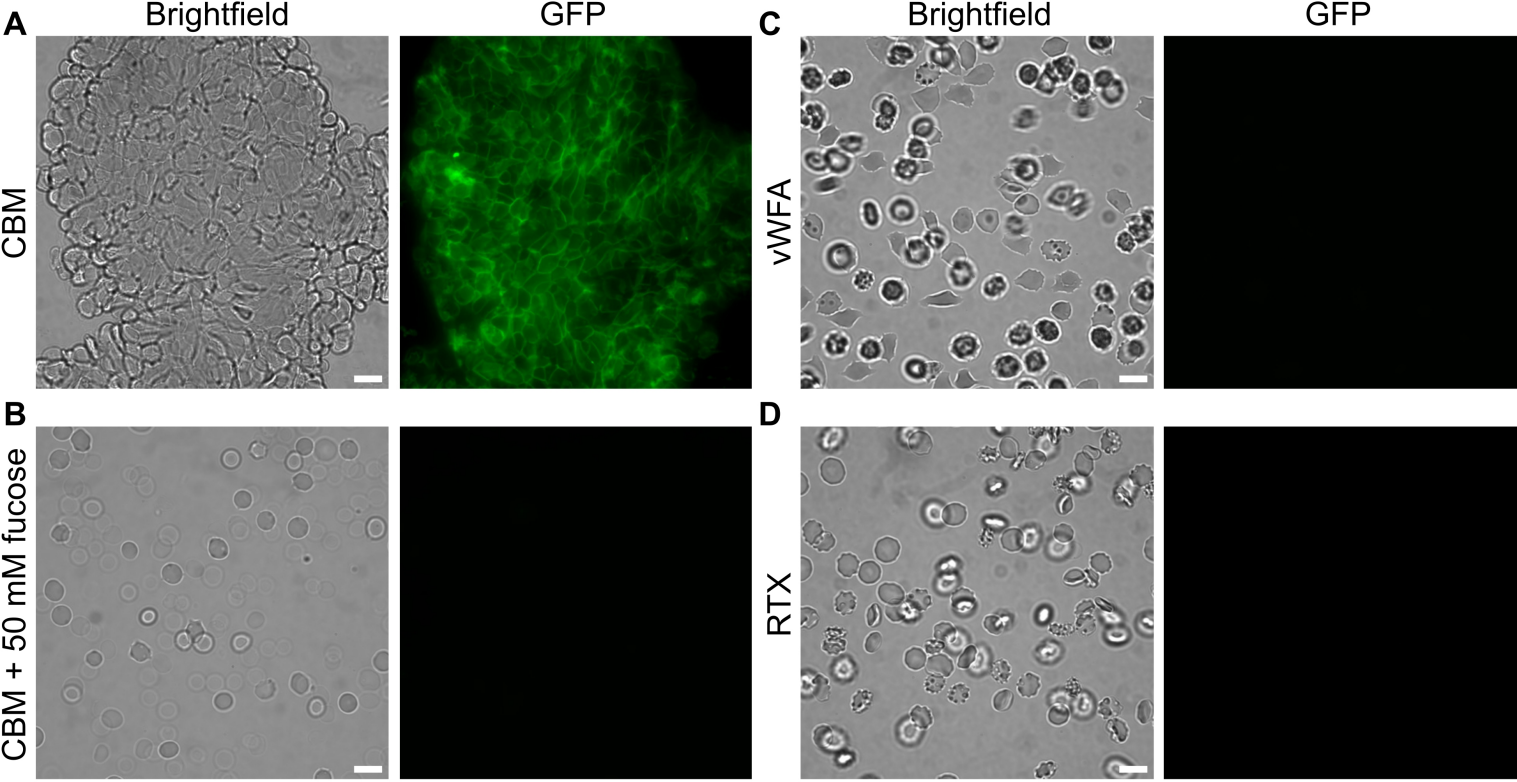
Binding of *Ah*LapCBM_3_ to human erythrocytes, with and without fucose present. (**A**) Erythrocytes incubated with GFP-tagged *Ah*LapCBM_3_ construct that includes a split domain and one extender domain (Fig. 3A through C). The left-hand panel is viewed in brightfield and the right-hand panel is viewed to see the green fluorescence of GFP. (**B**) Erythrocytes incubated with the same GFP-tagged *Ah*LapCBM_3_ construct in the presence of 50 mM fucose. (**C**) Erythrocytes incubated with the GFP-tagged *Ah*LapvWFA construct. (**D**) Erythrocytes incubated with the GFP-tagged *Ah*LapRTX construct. The white scale bar indicates 10 µm.

Having identified fucosylated glycans as a binding target for the *Ah*LapCBM_3_, we looked at this protein’s affinity for different cell types and biofilms. CBMs from RTX adhesins of *V. cholerae* and *A. veronii* also bound to fucosylated glycans that included some blood group antigens (35). When these CBMs were fluorescently labeled and incubated with human erythrocytes, they bound to these cells but also lysed them in a concentration-dependent manner. When the GFP-tagged *Ah*LapCBM_3_ prepared for glycan array analysis was incubated with human erythrocytes, it bound to the cells and aggregated them together into huge, tight, fluorescent clusters (Fig. 6A). However, it did not lyse them. When this experiment was repeated in the presence of 50 mM l-fucose, there was no binding of the CBM_3_ to erythrcytes and no aggregation of the cells (Fig. 6B), which is consistent with the binding interaction being mediated through a fucosyl moiety on the erythrocyte glycans. When the other two putative ligand-binding domains were GFP-tagged and mixed with human erythrocytes, they showed no affinity and no ability to cause aggregation of these cells (Fig. 6C and D).

**Fig 7:**
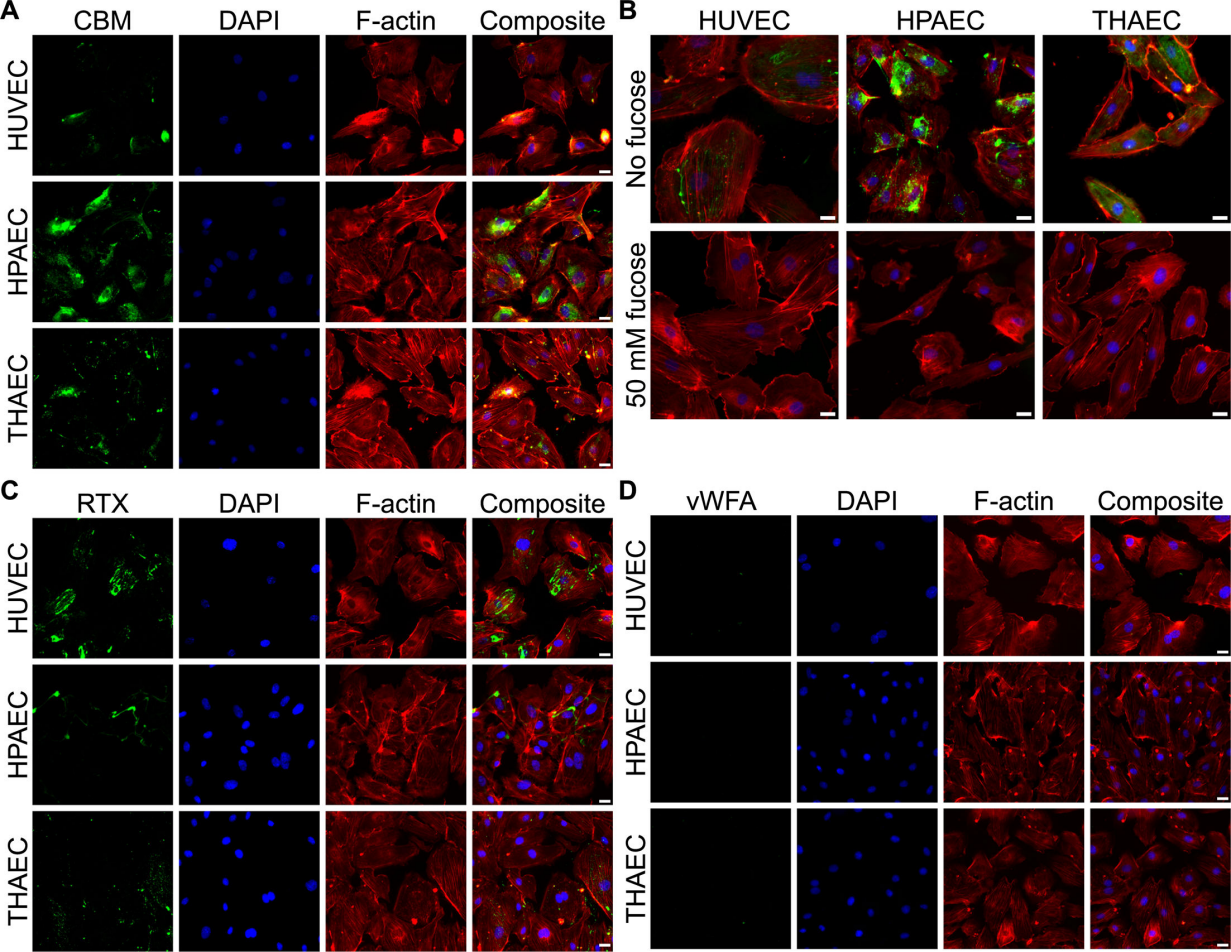
*Ah*LapCBM_3_ binding to endothelial cells in the absence and presence of fucose. (**A**) The three images in the first column show fluorescence from the incubation of GFP-tagged *Ah*LapCBM_3_ with human umbilical vein endothelial cells (HUVECs), human pulmonary artery endothelial cells (HPAECs), and telomerase-immortalized human aorta endothelial cells (THAECs). Images in the second column show the same cells stained with DAPI to visualize the nuclei. Images in the third column show the same cells stained with phalloidin to visualize the actin cytoskeleton. Images in the fourth column show composite images of the three staining patterns superimposed. (**B**) Composite staining images of the three endothelial cell types were repeated in the absence (top row) and presence (bottom row) of 50 mM fucose. The white scale bar indicates 10 µm. (**C**) The three images in the first column show fluorescence from the incubation of GFP-tagged *Ah*LapRTX with HUVECs, HPAECs, and THAECs. Images in the second column show the same cells stained with DAPI to visualize the nuclei. Images in the third column show the same cells stained with phalloidin to visualize the actin cytoskeleton. Images in the fourth column show composite images of the three staining patterns superimposed. (**D**) The 12 panels shown here are equivalent to those in (A) and (C), except that the cells in the first column were probed with GFP-tagged *Ah*LapvWFA.

To determine if the CBM_3_ can bind to other human cell types, three different types of endothelial cells, HUVEC (human umbilical vein endothelial cells), HPAEC (human pulmonary artery endothelial cells), and THAEC (telomerase-immortalized human aorta endothelial cells) were probed with GFP-labeled *Ah*LapCBM_3_. Extensive patches of fluorescence labeling were seen on the surface of all three cell types (Fig. 7A). For reference, the same cells were stained with DAPI to show the nuclei and phalloidin to visualize the actin cytoskeleton, with a superimposition of all three images shown to the right of each set. A similar superimposition of the three images for the three endothelial cell types was made in the absence (top) and presence of 50 mM fucose (bottom) (Fig. 7B). Fucose completely blocked all binding of the GFP-labeled *Ah*LapCBM_3_ to all three endothelial cell types.

### *Ah*LapRTX, but not *Ah*LapvWFA, has affinity for endothelial cells

When GFP-tagged *Ah*LapRTX was incubated with the same array of endothelial cells, it also bound to all three of them (Fig. 7C). It is not currently known what component of the endothelial cells is recognized and bound by this domain. When GFP-tagged *Ah*LapvWFA was incubated with the same endothelial cells, none of them were bound by this protein (Fig. 7D).

### Binding of *Ah*Lap ligand-binding domains to yeasts

**Fig 8:**
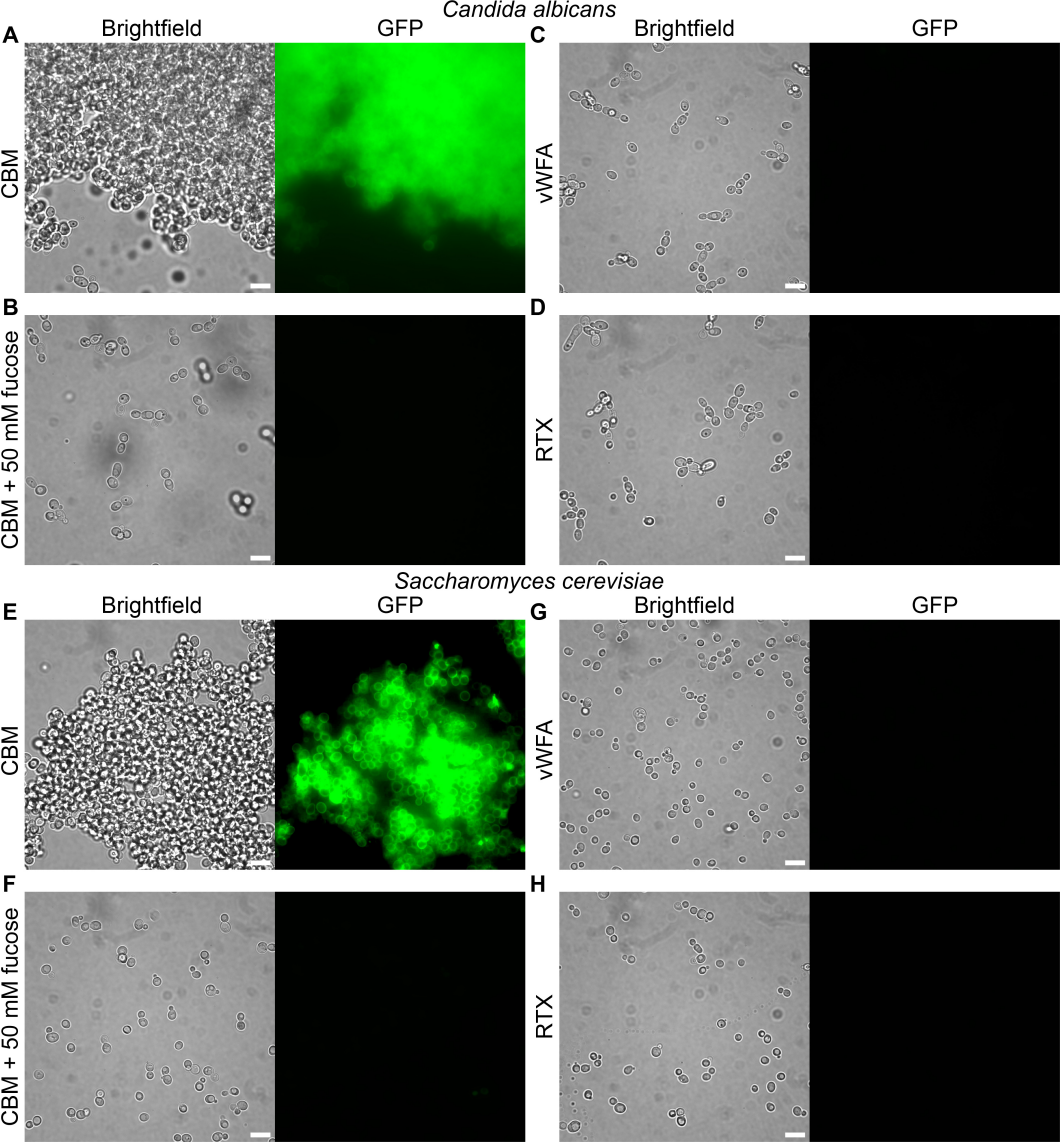
*Ah*LapCBM_3_ binding to yeasts in the presence and absence of fucose. (**A**) The two panels show *Candida albicans* incubated with GFP-labeled *Ah*LapCBM_3_ and visualized in brightfield (left) and fluorescence (right). **(B**) The two panels repeat the conditions in (A) but with 50 mM fucose present. Panels (**E**) and (**G**) are repeats of (A) and (B) with *Saccharomyces cerevisiae* instead of *C. albicans*. (**C**) The two panels show *C. albicans* incubated with GFP-labeled *Ah*LapvWFA and visualized in brightfield (left) and fluorescence (right). (**D**) The two panels show *C. albicans* incubated with GFP-labeled *Ah*LapRTX and visualized in brightfield (left) and fluorescence (right). Panels (**G**) and (**H**) are repeats of (C) and (D) with *S. cerevisiae* instead of *C. albicans*. The white scale bar indicates 10 µm.

When two yeast species, *Candida albicans* and *Saccharomyces cerevisiae*, were incubated with GFP-labeled *Ah*LapCBM_3_, both yeasts were intensively labeled (Fig. 8A and E). The addition of this three-domain construct induced aggregation of the yeasts into large cell clusters, very much like the aggregation of erythrocytes in Fig. 6A. This was despite the *S. cerevisiae* being a non-flocculating strain. To test for the presence of functional amyloids, the cell aggregates were stained with the amyloid-specific dye thioflavin T. Imaging by microscopy showed no increase in fluorescence by ThT (data not shown), suggesting an absence of amyloids. This contrasts with typical aggregation by *C. albicans* and *S. cerevisiae,* in which cell-to-cell contacts are mediated, in part, by functional amyloid formation (43). This, in turn, supports that the observed aggregation is mediated by *Ah*LapCBM_3_ rather than the yeasts’ native adhesins. Again, preincubation with 50 mM fucose blocked all fluorescent staining and aggregation, with the yeast dispersed as single cells (Fig. 8B and F). In contrast, the GFP-tagged *Ah*LapRTX and *Ah*LapvWFA domains showed no binding to either yeast species and no tendency to aggregate the yeast (Fig. 8C, D, G, H).

### Binding of *Ah*LapCBM_3_ to diatoms and biofilms

**Fig 9:**
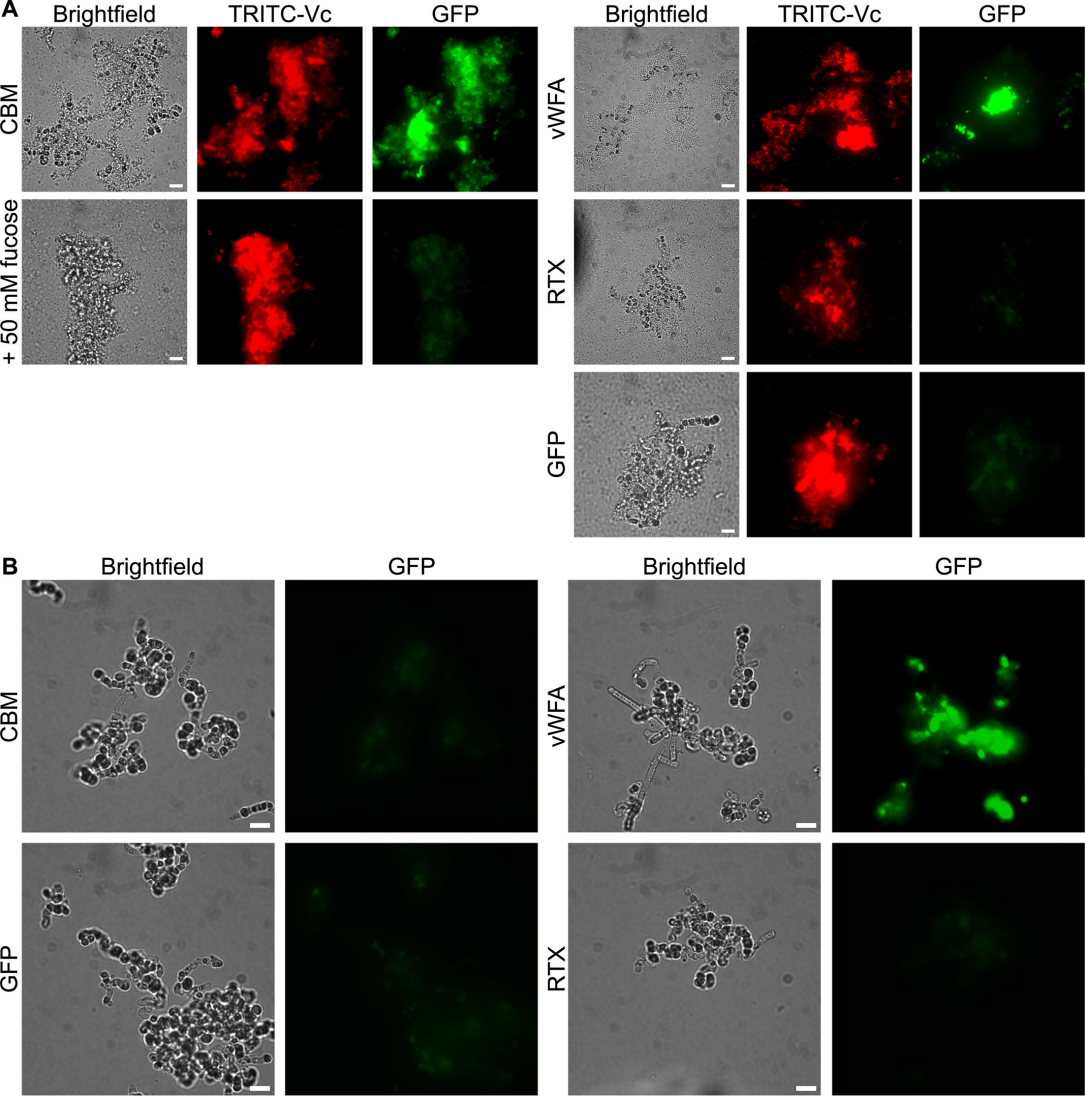
*Ah*Lap ligand-binding domain studies with diatoms, with and without *V. cholerae* present to generate biofilms. (**A**) The six panels on the left show binding of GFP-labeled *Ah*LapCBM_3_ to a co-culture biofilm formed by TRITC-labeled *V. cholerae* on *E. spinifer*. The left-hand column of panels shows the brightfield view of the co-culture. The middle column shows the red TRITC fluorescence of *V. cholerae* colonizing *E. spinifer*. The right-hand column shows the green GFP fluorescence of bound *Ah*LapCBM_3_. The top row of cells were incubated in the absence of fucose, and the bottom row of cells in the presence of 50 mM fucose. The nine panels on the right have the same horizontal sequence of brightfield, followed by TRITC staining to visualize *V. cholerae*, and GFP analysis. In the top sequence, the cells were incubated with GFP-labeled *Ah*LapvWFA. In the middle sequence, the cells were labeled with *Ah*LapRTX, and in the bottom sequence, the cells were mixed with free GFP to check for nonspecific labeling. (**B**) Here, diatoms in the absence of *V. cholerae* were incubated with the three GFP-tagged ligand-binding domains as indicated and with the GFP control. Brightfield and fluorescence images are compared side by side. The white scale bar indicates 10 µm.

Marine bacteria, like *V. cholerae,* use their adhesins to colonize a wide variety of organisms and microorganisms in their aqueous habitat (44). When *V. cholerae* encountered the diatom *Extubocellulus spinifer*, the bacteria bound to them and formed biofilms (Fig. 9A). The brightfield image shows several diatoms enmeshed in bacteria and the biofilm the bacteria produce in this situation. The bacteria were stained red with TRITC. These mixed species biofilms provided an opportunity to test *Ah*LapCBM_3_ for binding to all three components: diatoms, bacteria, and biofilm matrix. In the absence of *V. cholerae,* and hence the absence of biofilm, GFP-tagged *Ah*LapCBM_3_ failed to bind to the diatoms (Fig. 9B). However, after the addition of *V. cholerae*, *Ah*LapCBM_3_ bound to the zones of biofilm produced by the red fluorescently labeled bacteria around the diatoms (Fig. 9A). Fluorescence was particularly intense in some areas of the mixed species biofilm. There was no obvious binding to the free bacteria.

When GFP-labeled *Ah*LapCBM_3_ was added to the mix of bacteria and diatoms, its green fluorescence coincided with the locations of the red *V. cholerae*. Also, the most intense patches of green matched closely to those regions of high bacterial density. When these binding experiments were repeated in the presence of 50 mM fucose, biofilms were still formed, but the binding by GFP-tagged *Ah*LapCBM_3_ was almost eliminated. These results are consistent with *Ah*LapCBM_3_ binding to polysaccharides released by *V. cholerae* during biofilm formation.

### *Ah*LapvWFA binds to diatoms and biofilms

The widespread presence of vWFA domains in adhesins from diverse bacterial species, including the plant bacterium *Pseudomonas putida* (45), suggests that proteins other than those of the mammalian extracellular matrix can be their targets. For this reason, we tested GFP-labeled *Ah*LapvWFA for binding to all the same cells tested with the other two ligand-binding domains. There was no binding of the vWFA domain to the endothelial cells (Fig. 7D), to human erythrocytes (Fig. 6C), or to the two yeasts tested (Fig. 8C and G), but unlike the CBM_3_ and RTX domains, it did bind directly to the diatom *E. spinifer,* both in the absence and presence of biofilm-producing *V. cholerae* (Fig. 9A and B).

## DISCUSSION

As recently reported by Sherik et al*.* (35) and Graham et al. (36), AlphaFold is adept at folding the long chain of domains that make up RTX adhesins and at recognizing the split domains out of which ligand-binding domains protrude. In *Ah*Lap, there are a total of 42 domains of which three can be classified as ligand-binding. Expression constructs for these three domains were originally designed and synthesized prior to the advent of AlphaFold by relying on sequence alignments, homology modeling, and guesswork. AlphaFold3 is far superior at predicting the beginning and end of each domain but fortunately, these three constructs were not lacking any portions of domains needed for folding. In addition, the crystal structures for *Ah*LapRTX and *Ah*LapvWFA ligand-binding domains are in excellent agreement with the AlphaFold3 models (Fig. S2A and B).

The three ligand-binding domains of *Ah*Lap are markedly different from each other in their structures and range of targets that they adhere to. *Ah*LapCBM_3_, which has micromolar affinity for fucosylated glycans like the Lewis B and Y antigens, was the most promiscuous binder. It bound to human erythrocytes, a range of endothelial cells, and to yeasts, but not directly to the diatom *E. spinifer*. However, when *V. cholerae* was present and had a chance to form biofilm on the diatoms, *Ah*LapCBM_3_ was able to bind to this surface. The *V. cholerae* strain used to generate the biofilms has had both of its toxin genes eliminated so they posed no known health risks to the experimenters.

The other two ligand-binding domains in *Ah*Lap give this one RTX adhesin additional versatility in binding different targets. Thus, the vWFA domain was able to directly bind to the *E. spinifer* diatoms, which *Ah*LapCBM_3_ could not do. This additional interaction leads to biofilm formation, which helps the bacteria’s colonization of its aqueous habitats, protects it from predation by amoebas, and provides a way to evade host defenses during ingestion or breaching of the skin. There were no signs of *Ah*LapvWFA binding to yeasts, human erythrocytes, or endothelial cells. The RTX-like domain is positioned in *Ah*Lap as a ligand-binding module emerging from a split domain. It did not bind to any of the microorganisms tested, nor to the erythrocytes, but it did bind in patches to all three human endothelial cell types.

*Ah*LapRTX appears to be a novel ligand-binding domain, and there are as yet no indications of what macromolecules it might adhere to. In a recent survey of 11 RTX adhesins, there is one structural homolog present in an adhesin from *V. vulnificus* (34). These RTX ligand-binding domains are separate from the larger C-terminal RTX domain adjacent to the Type I secretion signal that has been suggested to initiate the folding of the adhesin during secretion into a Ca^2+^-rich environment (46). However, there is a report of an extended, segmented C-terminal RTX domain from an RTX toxin being used to bind fibronectin, which shows the potential of this fold to be involved in adhesion (47).

Once ligands are recognized for the different *Ah*Lap adhesive domains, it should be possible to develop antagonists to block binding. For example, millimolar concentrations of free fucose were effective in blocking the binding of *Ah*LapCBM_3_ to all of its targets (erythrocytes, endothelial cells, yeasts, and biofilms). The same strategy was previously used to block the CBMs of *V. cholerae* and *A. veronii* from binding and lysing human erythrocytes (35), and the CBM of *M. primoryensis* from binding the diatom *Chaetoceros neogracile* (30). In the situation where two or more ligand-binding domains are linked together, as they are in the adhesins attached to their bacterial host, it might be necessary to simultaneously provide antagonists to each of the ligand-binding domains to release their collective grip. An example mentioned previously, the *Mp*IBP adhesin, contains a PBD lying adjacent to the CBM which binds C-terminal tripeptides with the optimal sequence -Tyr-Thr-Asp (31). To release this adhesin section from *C. neogracile*, it has been necessary to simultaneously provide both fucose and the peptide antagonist (data not shown). *V. cholerae* and *M. primoryensis* are both examples where there is a single dominant adhesin at work in the strains examined. Other bacteria, like *P. fluorescens*, can simultaneously express two adhesins (48). In this situation, blocking bacterial binding will require a thorough analysis of all adhesins at play that might extend the niches and hosts colonized by the bacteria. In a situation where ligands cannot be found for a particular ligand-binding domain, it should be possible to still block adhesion by raising polyclonal antibodies to the domain. This was the case for the ice-binding domain of the *M. primoryensis* ice adhesin, where an antibody to this domain completely blocked the binding of the bacteria to ice (29).

This structure–function study on *Ah*Lap, which is one type of fibrillar adhesin from the pathogen *Aeromonas hydrophila*, illustrates the complexity of the interactions the bacteria might display in different stages of its life cycle and with the different hosts and surfaces that make up its niche. Bioinformatic strategies outlined here for recognizing RTX adhesins can quickly zero in on these structures in other Gram-negative bacteria, and the recognition of split domains by AI-driven folding programs can reveal the number and types of ligand-binding domains present (36). Identifying their ligands is not simple but offers the possibility of developing low molecular weight antagonists that can block or reduce colonization. However, colonization and infection by the bacteria could also be blocked by immunization through using the ligand-binding domains as antigens. These approaches offer a tailored tactic to combat specific bacterial pathogens that is unlikely to result in resistance and can complement the use of antibiotics as the latter wane in their effectiveness.

## MATERIALS AND METHODS

### Bioinformatics and protein modeling

The RTX adhesin *Ah*Lap sequence (UniProtKB ID: A0KNW4) was identified by database searches as described for RTX adhesins in general (36). *Ah*Lap was compared to itself using EMBOSS Dotmatcher (https://www.bioinformatics.nl/cgi-bin/emboss/dotmatcher) to generate a dot matrix analysis to locate repetitive sequences such as the BIg extender domains and the nonapeptide repeats that make up the C-terminal RTX beta-solenoid domain close to the C terminus.

The structure of the adhesin was predicted by AlphaFold3 (49). This allowed the *Ah*Lap domain map to be compared alongside the dot matrix analysis. The model for the entire adhesin was rendered in PyMOL V2.5.2 (Schrödinger Inc.) and examined to reveal split domains that indicate associated ligand-binding domains.

### Cloning and expression of *Ah*Lap ligand-binding domains

DNA sequences encoding the carbohydrate-binding construct (*Ah*LapCBM_3_) (residues ^3455^NDAP-----AAEG^3940^), RTX-beta-roll domain (RTX) (residues ^4168^AVAD---- VLPG^4498^), and von Willebrand factor A domain (vWFA) (^4497^PGQN----NNLP^4776^) were synthesized by GeneArt (Life Technologies) to allow codon optimization for expression in *E. coli*. The DNA constructs were ligated into the pET28a expression vector, which provides an N-terminal hexahistidine tag. Positive clones were confirmed by DNA sequencing and transformed into *E. coli* BL21 (DE3) cells for protein production.

Single colonies of the clones were each picked into 25 mL cultures of LB broth +0.1 mg/mL kanamycin and grown overnight at 37 °C. Overnight cultures were used to inoculate 1 L cultures, which were then grown at 37 °C until an OD_600_ of 1.0 was reached. Isopropyl β-d-1-thiogalactopyranoside was added to a final concentration of 1 mM to induce protein expression of *Ah*LapCBM_3_ and *Ah*LapRTX, and 0.4 mM to induce expression of *Ah*LapvWFA, respectively, at 20 °C. The cultures were grown overnight.

### Purification of *Ah*Lap ligand-binding domains

Induced cells were pelleted in a JS4.2 rotor (Beckman Coulter) at 4,600 × *g* for 30 min at 4 °C. The cell pellets were resuspended in 50 mL of 50 mM Tris-HCl (pH 9.0), 500 mM NaCl, 2 mM CaCl_2_, and 0.1 mM phenylmethylsulfonyl fluoride (lysis buffer). Cells were lysed by sonication, and the cell debris was removed through centrifugation in a JA25.5 rotor (Beckman Coulter) at 50,750 × *g* for 40 min. The supernatant was loaded onto a Ni-NTA affinity chromatographic column (Qiagen) in 50 mM Tris-HCl (pH 9.0), 500 mM NaCl, and 2 mM CaCl_2_. After washing the column in the same buffer supplemented with 5 mM imidazole, the bound protein was eluted with this buffer containing 400 mM imidazole. Fractions enriched in target proteins were subjected to size-exclusion chromatography on a Superdex 200 Increase 10/300 column (GE Healthcare). For *Ah*LapCBM_3_, the peak fractions were re-loaded onto a Ni-NTA column for further purification, and the eluted peak was dialyzed against 20 mM Tris-HCl (pH 9.0) overnight at 4 °C. The peak fractions of *Ah*LapRTX from size-exclusion chromatography were dialyzed in 50 mM Tris-HCl (pH 9.0) (buffer A) overnight and subjected to Q-Sepharose ion-exchange chromatography on a High Performance HiLoad 16/10 column (GE Healthcare) to elute proteins with a linear gradient of buffer B containing 50 mM Tris-HCl (pH 9.0) and 1 M NaCl. The purified *Ah*LapRTX was dialyzed against 50 mM NaCl and 20 mM Tris-HCl (pH 7.6), overnight. All three recombinant proteins were checked for purity by SDS-PAGE with Coomassie blue staining before they were concentrated, aliquoted, flash-frozen, and stored at −80 °C until further use.

### Protein crystallization

Initial crystallization trials with *Ah*LapRTX and *Ah*LapvWFA were done by sitting-drop vapor-diffusion and microbatch-under-oil methods with various crystallization screen kits (Qiagen) at 295 K. Only conditions that were compatible with millimolar Ca^2+^ concentrations were used. Thin multi-plate crystals of *Ah*LapRTX (5 mg/mL) grew in precipitant condition of 20% (w/v) PEG 8000, 0.1 M MES (pH 6.0), and 0.2 M calcium acetate. Single crystals of *Ah*LapRTX were obtained by adding 10% v/v of Tacsimat (pH 7.0) and 0.02 M HEPES-NaOH (pH 6.8) from the Silver Bullets library of additives (Hampton Research) to the above crystallization condition. The crystals of *AhLap*-vWFA (10 mg/mL) grew in 20% PEG 3350 (w/v) and 0.2 M CaCl_2_. All diffraction-size crystals were flash-frozen in liquid nitrogen with a cryoprotectant that included crystallization solution plus 30% ethylene glycol or 30% glycerol or 40% PEG 400, respectively, for the two different protein crystals. Since there were no homologous structures in the RCSB PDB that corresponded to the amino acid sequence of *Ah*LapRTX, native crystals were soaked in 0.7 mM HoCl_3_ in its cryoprotectant for heavy atom derivatization for phasing. Crystals were soaked in the solution for approximately 1–10 min before flash freezing in liquid nitrogen.

### Structure determination

Single-wavelength calcium anomalous X-ray diffraction data for *Ah*LapvWFA crystals were collected at 100 K on the home source with a wavelength of 2.29 Å using a Rigaku MicroMax-007 HF rotating-anode X-ray generator equipped with a chromium target and an R-AXIS IV++ image-plate detector. Native data for *Ah*LapvWFA crystals were collected at a wavelength of 1.03318 Å on the 23ID-B beam line at the Argonne National Laboratory (Advanced Photon Source, Lemont, USA). For HoCl_3_-soaked derivatized crystals of *Ah*LapRTX, single-wavelength anomalous diffraction data were collected at a wavelength of 1.5348 Å, and native crystal data sets were collected at a wavelength of 0.9775 Å at 100 K on the CLSID08-1 beamline at the Canadian Light Source (Saskatoon, Canada). All diffraction image datasets from the two proteins were indexed and integrated with XDS (50) and then scaled with AIMLESS in CCP4 (51). AutoSol from the PHENIX suite (52) was used to identify Ca-atom and Ho-atom anomalous substructure sites as well as to calculate phases. After getting phasing solutions, the AutoBuild (53, 54) was then applied to build and refine the initial models. To increase the resolution of the *Ah*LapvWFA structure, the Molecular Replacement method with PHASER in the CCP4 suite (55) was performed with synchrotron data, and a low-resolution structure solved on the home source was used as a model template. Subsequent structure building for the two proteins was done by iterative manual model building using COOT (56) with refinement using PHENIX (57) and REFMAC5 (58). Figures were generated in PyMOL V2.5.2 (Schrödinger Inc.). Crystallographic data collection and refinement statistics are summarized in Tables S1 and S2.

### Addition of fluorescent tags to ligand-binding domains and to bacteria

All three ligand-binding domains, *Ah*LapCBM_3_, *Ah*LapRTX, and *Ah*LapvWFA, were fluorescently tagged by inserting the GFP gene sequence in frame at the 5ʹ-end of their gene sequences. The GFP-tagged proteins were produced and purified as described above for the non-tagged proteins.

*V. cholerae* classical strain, with both toxin genes deleted, was grown overnight at 37 °C in 5 mL of LB Miller media. Bacteria were pelleted by centrifugation at 10,000 × *g* for 5 min and resuspended in 10 mL of seawater/F2 media with 10 µL of TRITC from a 10 mg/mL stock (Invitrogen) added for 15 min. Bacteria were pelleted again, and the supernatant was removed and discarded. The cells were resuspended in seawater/F2 media (5 mL) and washed one time by another cycle of pelleting and resuspension as described above.

### Glycan array analysis

Mammalian glycan array version 5.4 was screened with *Ah*LapCBM_3_ and *Ah*LapRTX by the Consortium for Functional Glycomics (Harvard Medical School). This printed glycan array consisted of 585 mammalian glycans that each appeared six times. The array was incubated with GFP-tagged *Ah*LapCBM_3_ and *Ah*LapRTX at two different concentrations, 5 and 50 µg/mL. The green fluorescence of the fusion protein was used to measure the relative fluorescence units (RFU) of the bound protein. The highest and lowest values from each set of six replicates were discarded to remove outliers, and an average value of the remaining four replicates was used in the comparisons.

### Isothermal titration calorimetry

Isothermal titration calorimetry (ITC) measurements for *Ah*LapCBM_3_ binding Lewis B antigen were done using a MicroCal iTC200 calorimeter (Malvern) set at 30 °C. *Ah*LapCBM_3_ was dialyzed overnight against 50 mM Tris-HCl (pH 9.0), 200 mM NaCl, and 2 mM CaCl_2_ and then diluted to different concentrations from 170 to 467 µM in the same buffer. The final thermogram was produced using 370 µM. Lewis B antigen (Biosynth) solution at a concentration of 3.55 mM in the same buffer was added as 5 µL aliquots into the *Ah*LapCBM_3_ solution (350 µL) at 5 min intervals from a computer-controlled syringe rotating at 400 rpm. After a total of 30 injections, the data were analyzed by Origin V5.0 (Malvern).

ITC measurements for *Ah*LapCBM_3_ binding free fucose were done using a MicroCal VP-ITC calorimeter (Malvern) set at 30 °C. *Ah*LapCBM_3_ was dialyzed overnight against 50 mM Tris-HCl (pH 9.0), 200 mM NaCl, and 2 mM CaCl_2_ and then diluted to different concentrations in the same buffer. The final thermogram was produced using 323 µM. Free fucose (Fisher Scientific, Cat # F006510G) solution at a concentration of 6.5 mM in the same buffer was added as 10 µL aliquots into the *Ah*LapCBM_3_ solution (1.5 mL) at 5 min intervals from a computer-controlled syringe rotating at 400 rpm. After a total of 30 injections, the data were analyzed by Origin V7.0 (Malvern).

### Erythrocyte and endothelial cell binding experiments

Type-O blood was obtained from ZenBio (NC, USA). Erythrocytes were prepared by pelleting cells at 1,000 × *g* in Krebs–Henseleit solution (KRT) buffer (120 mM NaCl, 5 mM KCl, 1 mM MgSO_4_, 3 mM CaCl_2_, 10 mM Tris-HCl, pH 7.4). Erythrocytes were washed an additional three times in KRT buffer and resuspended to 10% (v/v) in KRT buffer. Erythrocyte binding experiments were done using GFP-tagged *Ah*LapCBM_3_ at a concentration of 9.5 µM. The labeled protein was incubated with 1.4% (v/v) erythrocytes in KRT buffer for 30 min. Erythrocytes were then pelleted by centrifugation for 3 min at 4,500 × *g*, and the supernatant was discarded. This procedure was repeated three times to wash out unbound GFP-tagged *Ah*LapCBM_3_ before the erythrocyte pellet was finally resuspended in 20 µL of buffer and examined on slides by fluorescence microscopy. Parallel experiments were performed to test if l-fucose could release GFP-tagged *Ah*LapCBM_3_ that was already bound to the erythrocytes. Here GFP-tagged *Ah*LapCBM_3_ was incubated with erythrocytes and 50 mM of l-fucose. The remainder of the experiment followed the same procedure as described above. Images were obtained using an Olympus IX83 inverted fluorescence microscope equipped with an Andor Zyla 4.2 Plus camera.

Red fluorescent protein-expressing HUVECs were obtained from Angio-Proteomie, Boston, MA. THAECs and HPAECs were a generous gift from Dr. Mark Ormiston, Queen’s University. Cells were cultured in Endothelial Growth Medium (EGM-2, Lonza, Switzerland) and were first grown to 80% confluence and then transferred to a 24-well plate with 12 mm circular glass cover at 2 × 10^4^ cells/mL. The cells were maintained in a humidified incubator at 37 °C and 5% CO_2_. Experiments were conducted after 20 h of growth. Cells were incubated with 0.1 mg/mL of protein for 30 min before washing three times with phosphate-buffered saline (PBS) followed by a 5-min incubation with 2% paraformaldehyde. Fixed cells were washed three times with PBS before staining F-actin for 45 min with TRITC-labelled phalloidin. Cells were washed three more times with PBS then with water before mounting on slides with coverslips and 5 µL of Fluoromount-G with DAPI (Invitrogen). Imaging was performed on an Olympus IX83 inverted fluorescence microscope or a Leica Mica confocal microscope. ImageJ (https://imagej.net/ij) was used to create composite images of the individual channels.

### Yeast binding experiments

Fungal strains used in this study are listed in Table S4. Growth and manipulation of *C. albicans* and *S. cerevisiae* followed basic procedures for budding yeast cultures. All strains were maintained on YPD plates containing 1% yeast extract (BD-Bacto), 2% peptone (BD-Bacto), and 2% glucose (BioShop). Experimental cultures were inoculated the night before in 10 mL of YPD culture and grown overnight at 30 °C. The overnight culture was used to inoculate fresh YPD for growth to the mid-logarithmic phase the next morning. Prior to performing the experiment, cultures were diluted to 1.0 × 10^5^ cells/mL. Yeast cells in a 1 mL aliquot of the culture were pelleted and resuspended in 1 mL of Tris-buffered saline (TBS; 50 mM Tris-HCl, 150 mM NaCl, pH 7.6) containing *Ah*LapCBM, *Ah*LapvWFA, or *Ah*LapRTX in the presence or absence of 50 mM fucose. The reactions were incubated overnight at room temperature and visualized by microscopy. Following visualization, thioflavin T (Thermo Scientific Chemicals, Cat# 211760050) suspended in TBS was added to each reaction to a final concentration of 100 nM. The samples were incubated for 30 min at 30 °C and visualized by microscopy.

### Diatom culturing

The diatom *Extubocellulus spinifer* was cultured in Gulf of Maine sea water (Bigelow National Center for Algae and Microbiota, East Boothbay, USA) supplemented with Guillard’s (F/2) marine water enrichment solution (Sigma-Aldrich, Cat # G0154) as previously described (34). Diatom cultures were grown at 20 °C shaking at 70 µE/m^2^⋅s and split 1:1 weekly.

### Biofilm formation

*V. cholerae* classical strain with both cholera toxin genes deleted (a generous gift from Dr. Karl Klose, University of Texas at San Antonio) was cultured in 5 mL LB broth overnight at 37 °C with shaking (34). The culture was pelleted at 4000 × *g* and the medium was replaced with 1 mL Gulf of Maine sea water supplemented with Guillard’s (F/2) marine water enrichment solution. Diatom cultures (25 mL) were also pelleted at 4,000 × *g* and resuspended in 1 mL of seawater medium. The diatom suspension (100 µL) and the *V. cholerae* suspension (100 µL) were mixed and incubated at 20 °C for 2 days. At this time, the top 100 µL of the mixed culture was removed, and 50 µL of 1 mg/mL fluorescently tagged protein was added and incubated for 18 h at 20 °C in the dark. Cultures were pelleted at 1,500 × g for 2 min and rinsed by resuspension in 500 µL of TBS before being pelleted again. This washing procedure was repeated one more time to remove any unbound fluorescently tagged protein. The final pellet was resuspended in 50 µL of TBS and imaged on an Olympus IX83 inverted fluorescence microscope using the 100× oil objective.

## Data Availability

Crystallographic data for the *Ah*LapRTX (PDB ID: 9CSE) and *Ah*LapvWFA (PDB ID: 9DAS) domains have been deposited in the Protein Data Bank. Additional data are available upon request.

## SUPPLEMENTAL MATERIAL

Figure S1 mbio.03158-24-s0001.pdf Size-exclusion chromatography of AhLapCBM_3_.

Figure S2 mbio.03158-24-s0002.pdf AlphaFold3 models of AhLap ligand-binding domains overlaid on corresponding crystal structures.

Supplemental Tables mbio.03158-24-s0003.docx Tables S1 to S4.

ASM does not own the copyrights to Supplemental Material that may be linked to, or accessed through, an article. The authors have granted ASM a non-exclusive, world-wide license to publish the Supplemental Material files. Please contact the corresponding author directly for reuse.
