# Supplementary material for: Aeromonas hydrophila RTX adhesin has three ligand-binding domains that give the bacterium the potential to adhere to and aggregate a wide variety of cell types
Source: mBio. 2025 Apr 17;16(5):e03158-24. doi: 10.1128/mbio.03158-24 (PMC12077191; doi:10.1128/mbio.03158-24)
Supplement: Figure S1 — Size-exclusion chromatography of AhLapCBM3. [file mbio.03158-24-s0001.pdf]

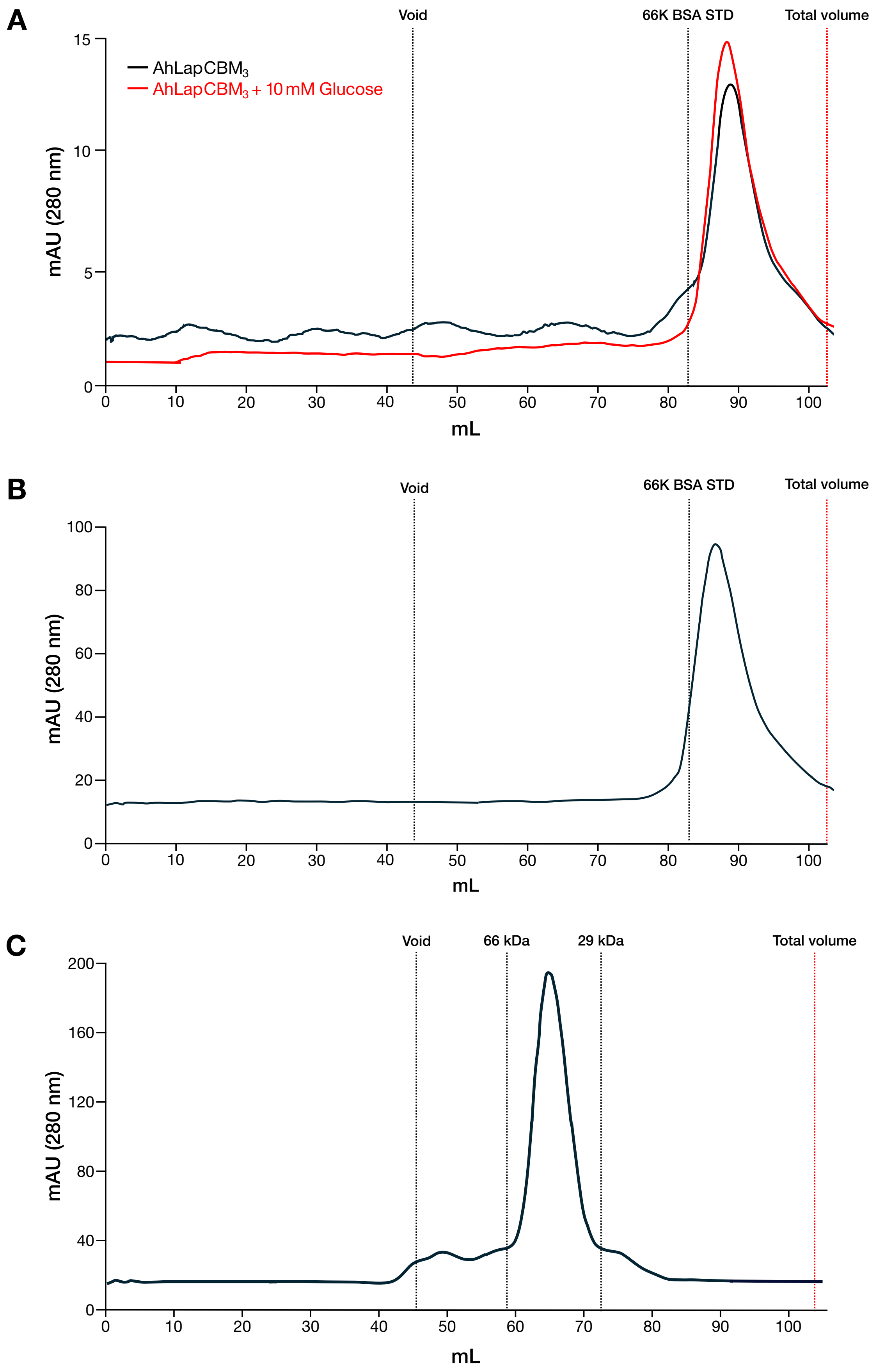

Figure S1. A) Size-exclusion chromatography of *Ah*LapCBM$_3$ in the absence (black profile) or presence (red profile) of 10 mM glucose. The void and total volume peaks of the column are marked by vertical dotted lines, as is the elution volume of the 66-kDa bovine serum albumin (BSA) standard. **B)** Size-exclusion chromatography of *Ah*LapvWFA domain on the same column with the same elution reference points. **C)** Size-exclusion chromatography of *Ah*LapCBM$_3$ on a calibrated S-75 column with molecular weight standards of 66 kDa and 29 kDa on either side of the *Ah*LapCBM$_3$ elution peak.
