# Supplementary material for: Aeromonas hydrophila RTX adhesin has three ligand-binding domains that give the bacterium the potential to adhere to and aggregate a wide variety of cell types
Source: mBio. 2025 Apr 17;16(5):e03158-24. doi: 10.1128/mbio.03158-24 (PMC12077191; doi:10.1128/mbio.03158-24)
Supplement: Figure S2 — AlphaFold3 models of AhLap ligand-binding domains overlaid on corresponding crystal structures. [file mbio.03158-24-s0002.pdf]

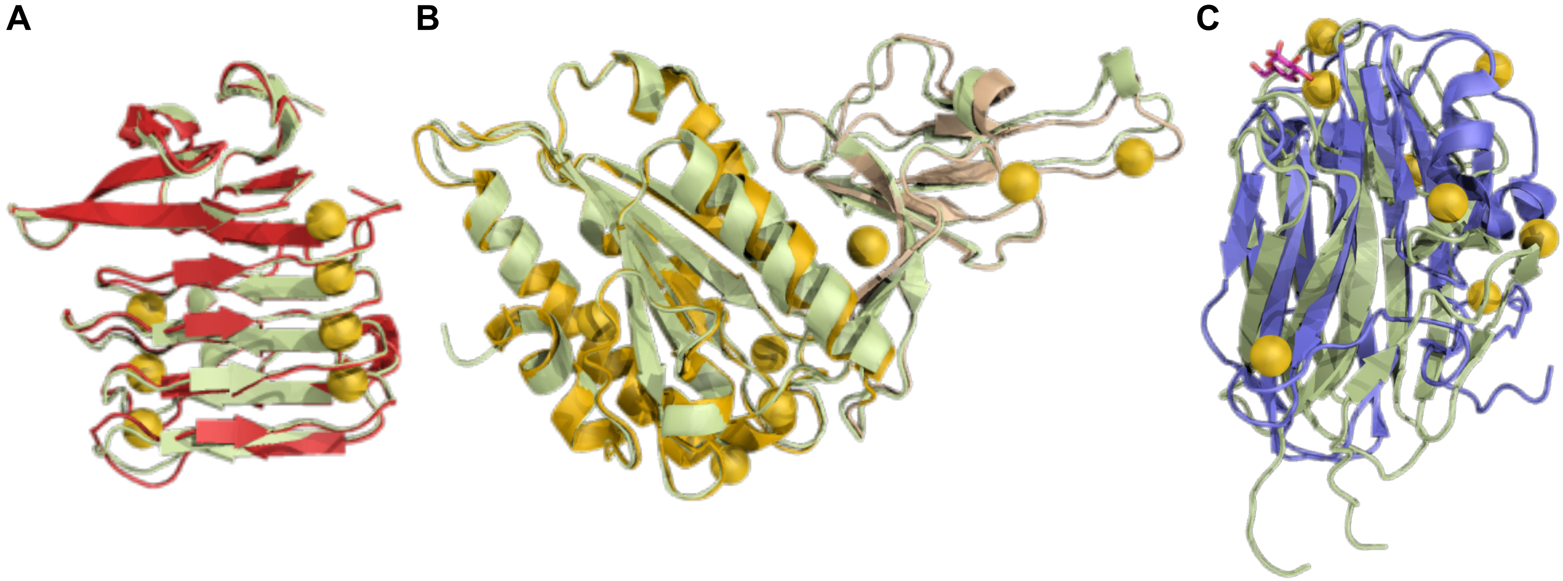

Figure S2. AlphaFold3 models of *Ah*Lap ligand-binding domains overlaid on corresponding crystal structures. The models are shown in green ribbon format and the domain colouring follows the format established in **Figure 1**. Ca^{2+} ions are represented as gold spheres. **A)** Model superimposed on the RTX ligand-binding domain shown in red. **B)** Model superimposed on the vWFA domain (gold) with the insertion sequence (sand). **C)** Model superimposed on the crystal structure of the CBM from *Mp*IBP (PDB ID: 5J6Y) shown in blue. Glucose (two overlapping anomers, α- and β-) is shown in pink. RMSD = 6.19 Å.
