# Supplementary material for: Aeromonas hydrophila RTX adhesin has three ligand-binding domains that give the bacterium the potential to adhere to and aggregate a wide variety of cell types
Source: mBio. 2025 Apr 17;16(5):e03158-24. doi: 10.1128/mbio.03158-24 (PMC12077191; doi:10.1128/mbio.03158-24)
Supplement: Supplemental Tables — Tables S1 to S4. [file mbio.03158-24-s0003.docx]

Table S1. Crystallographic data collection and refinement statistics for the *Ah*LapRTX domain

| **Data sets** | **Native** | **Holmium derivative** |
| --- | --- | --- |
| **Data collection statistics** | | |
| Beam sources | Synchrotron CLS08ID-1 | Synchrotron CLS08ID-1 |
| Wavelength (Å) | 0.97949 | 1.5384 |
| Space group | *C*222_1_ | *C*222_1_ |
| Unit cell dimensions (Å)  and angle (°) | *a*=67.60, *b*=103.33, *c*= 95.71  *α* = *β* = *ϒ* = 90 | *a*=67.97, *b*=101.68, *c*= 95.30  *α* = *β* = *ϒ* = 90 |
| Unique reflections | 24829 | 9480 |
| Resolution range (Å)* | 48.8 - 1.95 (2.0-1.95) | 56.58 - 2.7 (2.83 - 2.7) |
| *R_merge_* (%)* | 0.127 (1.66) | 0.69 (4.51) |
| *R_meas_* (%)* | 0.132 (1.72) | 0.72 (4.72) |
| CC 1/2* | 0.999 (0.73) | 0.968 (0.608) |
| < I/σ > (I) >* | 15.9 (2.0) | 7.1 (2.6) |
| Completeness (%)* | 100 (100) | 100 (100) |
| Redundancy* | 12.9 (12.9) | 11.2 (11.9) |
| **Refinement** | |  |
| Resolution (Å) | 48.75 - 1.95 |  |
| Number of reflections | 23607 |  |
| *R_work_*/*R_free_* | 0.203/0.258 |  |
| ***No. of atoms*** | |  |
| Protein | 2103 |  |
| Ca ion | 9 |  |
| Water | 226 |  |
| ***B-factors (Å^2^)*** | |  |
| Protein | 37.11 |  |
| CA ion | 40.81 |  |
| Water | 46.26 |  |
| ***Root mean square deviations*** | |  |
| Bond length (Å) | 0.012 |  |
| Bond angle (°) | 1.48 |  |
| ***Ramachandran plot statistics (%)*** | |  |
| Most favoured region | 97 |  |
| Additionally allowed regions | 3 |  |
| **PDB accession ID** | 9CSE |  |

* The numbers in parentheses are for the highest resolution shell.

Table S2. Crystallographic data collection and refinement statistics for the *Ah*LapvWFA domain

| **Data sets** | **Native** | **Holmium derivative** |
| --- | --- | --- |
| **Data collection statistics** | | |
| Beam source | APS 23ID_D | Home |
| Wavelength (Å) | 1.03318 | 2.29 |
| Space group | *C*1 2 1 | *C*1 2 1 |
| Unit cell dimensions (Å)  and angle (°) | *a*=71.24, *b*=156.29, *c*=57.50  *α=ϒ=* 90, *β*= 99.23 | *a*=71.39, *b*=156.67, *c*=57.55  *α=ϒ*= 90, *β*= 99.33 |
| Unique reflections | 119428 | 20818 |
| Resolution range (Å)* | 45.97 - 1.40 (1.45-1.40) | 46.05 - 2.5 (2.6-2.5) |
| *R_merge_* (%)* | 0.07 (1.87) | 0.06 (0.09) |
| *R_meas_* (%)* | 0.08 (2.01) | 0.06 (0.1) |
| CC 1/2* | 1.0 (0.49) | 0.998 (0.995) |
| < I/σ > (I) >* | 18.83 (1.24) | 33.6 (21.2) |
| Completeness (%)* | 98.45 (96.48) | 96.8 (93.4) |
| Redundancy* | 7.6 (7.4) | 7.4 (7.3) |
| **Refinement statistics** | |  |
| Resolution (Å) | 45.97 -1.403 |  |
| Number of reflections | 112830 |  |
| *R_work_*/*R_free_* | 0.19/0.21 |  |
| ***Number of atoms*** | |  |
| Protein | 4032 |  |
| CA ion | 12 |  |
| Water | 262 |  |
| ***B-factors (Å^2^)*** | |  |
| Protein | 22.2 |  |
| CA ion | 20.1 |  |
| Water | 26.38 |  |
| ***Root mean square deviations*** | |  |
| Bond length (Å) | 0.006 |  |
| Bond angle (°) | 0.808 |  |
| ***Ramachandran plot statistics (%)*** | |  |
| Most favoured region | 99 |  |
| Additionally allowed regions | 1 |  |
| **PDB accession ID** | 9DAS |  |

* The numbers in parentheses are for the highest resolution shell.

Table S3. Comparison of crystallographic and modeled structures for the ligand-binding domains of *Ah*Lap

| **Region** | **Residues** | **RMSD (Å)** |
| --- | --- | --- |
| von Willebrand Factor A | 4491 – 4777 | 0.564 (chain A)  0.479 (chain B) |
| Beta-roll domain | 4186 – 4350 | 0.357 |

Table S4. Names, genotypes, and sources of strains used in this study

| **Strain** | **Organism** | **Genotype** | **Source/parent** |
| --- | --- | --- | --- |
| CF026 | *Candida albicans* (1, 2) | *MATa his1^-^/ his1^-^ leu2^-^/ leu2^-^ arg4^-^/ arg4^-^* | RBY1133 |
| YMD963 | *Saccharomyces cerevisiae* (3) | *MATa leu2d0*  *ura3d0 his3-1 met15d0 LYS2+* | S288C |

**References for Supplementary Material**

1. Frazer C, Joshi M, Delorme C, Davis D, Bennett RJ, Allingham JS. 2015. *Candida albicans* Kinesin Kar3 Depends on a Cik1-Like Regulatory Partner Protein for Its Roles in Mating, Cell Morphogenesis, and Bipolar Spindle Formation. Eukaryot Cell 14:755-74.

2. Sherwood RK, Bennett RJ. 2008. Microtubule motor protein Kar3 is required for normal mitotic division and morphogenesis in *Candida albicans*. Eukaryot Cell 7:1460-74.

3. Neville N, Lehotsky K, Yang Z, Klupt KA, Denoncourt A, Downey M, Jia Z. 2023. Modification of histidine repeat proteins by inorganic polyphosphate. Cell Rep 42:113082.
